# Exploring super-resolution spatial downscaling of several meteorological variables and potential applications for photovoltaic power

**DOI:** 10.1038/s41598-024-57759-8

**Published:** 2024-03-27

**Authors:** Alessandro Damiani, Noriko N. Ishizaki, Hidetaka Sasaki, Sarah Feron, Raul R. Cordero

**Affiliations:** 1 NIES, Tsukuba, Japan; 2 University of Groningen, Leeuwarden, The Netherlands; 3 Universidad de Santiago de Chile, Santiago, Chile

**Keywords:** Climate sciences, Environmental sciences, Energy science and technology

## Abstract

We applied a perfect prognosis approach to downscale four meteorological variables that affect photovoltaic (PV) power output using four machine learning (ML) algorithms. In addition to commonly investigated variables, such as air temperature and precipitation, we also focused on wind speed and surface solar radiation, which are not frequently examined. The downscaling performance of the four variables followed the order of: temperature > surface solar radiation > wind speed > precipitation. Having assessed the dependence of the downscaling accuracy on the scaling factor, we focused on a super-resolution downscaling. We found that the convolutional neural network (CNN) generally outperformed the other linear and non-linear algorithms. The CNN was further able to reproduce extremes. With the rapid transition from coal to renewables, the need to evaluate low solar output conditions at a regional scale is expected to benefit from CNNs. Because weather affects PV power output in multiple ways, and future climate change will modify meteorological conditions, we focused on obtaining exemplary super-resolution application by evaluating future changes in PV power outputs using climate simulations. Our results confirmed the reliability of the CNN method for producing super-resolution climate scenarios and will enable energy planners to anticipate the effects of future weather variability.

## Introduction

Climate simulations based on general circulation models (GCMs) are critical for building adaptation plans and climate resilience strategies for food, energy, water, and renewables and reducing vulnerability/risks. However, GCMs often show systematic biases compared to observations, which must be corrected prior to their application in impact studies^1,2^. Furthermore, their projections cannot be directly used for regional applications due to their broad spatial resolution.

Downscaled simulations can be obtained by running nested simulations using the original climate data as boundary conditions^3,4^. However, this dynamical downscaling approach is computationally expensive and cannot be applied extensively.

Statistical downscaling approaches use empirical methods to increase the resolution of the original data within a reasonable time frame^5^. Due to recent interest in machine learning (ML), several studies have focused on developing a perfect prognosis approach to train ML models with historical climate datasets and using the learned relationship to downscale future simulations^6,7^. The method assumes that GCMs must realistically simulate predictors and the trained ML models remain effective under future climate scenarios. This approach often adopts perfect predictors (i.e., reanalysis) to reproduce the variability of high-resolution observations (i.e., predictands), allowing isolation of the benefits and drawbacks of downscaling. A further significant advantage is that newly-constructed ML models are directly portable to several GCMs. Nevertheless, the capability of ML techniques requires further investigation because some remaining issues prevent their broad adoption. ML approaches often consist of a black box method, making it difficult to reproduce the results^8^.

Many previous studies have adopted ML to downscale climate simulations and observations, sometimes outperforming more traditional methods^9,10^. Both ensemble methods (e.g., random forest, RF) and artificial neural network (ANN) algorithms (e.g., multi-layer perceptron regressor or deep learning) can reproduce non-linear relationships and have rapid and efficient applicability. Artificial neural networks are used more frequently because they do not demand accurate selection of the best set of predictors, as required for other algorithms such as multiple linear regression (MLR).

In the traditional perfect prognosis approach, the ML model is trained and applied independently to each spatial domain grid cell. Although its application is much faster than any dynamical downscaling approach, it remains time-consuming and causes any spatial information from neighboring grid cells to be lost. It may be possible to reduce spatial discontinuity by including neighboring cells as additional predictors or using the leading principal components of the predictors in a given region^5^. However, these approaches introduce undesirable complexity to the ML model.

To overcome this problem, recent research has been conducted based on applying methods developed from image processing techniques, such as convolutional neural networks (CNNs), which apply several convolution filters to successively learn image features to reduce image complexity. Usually, a CNN consists of convolutional layers followed by dense, fully connected feed-forward layers. Vandal et al.^11^ were among the first researchers to suggest the application of CNNs for downscaling observations. Later, Bano Medina et al.^12^ applied a relatively simple CNN architecture to downscale observations over Europe with better accuracy than traditional methods. Recently, more sophisticated CNN techniques have focused on precipitation^13^ and wind^14^. Other recent studies have conducted high-resolution downscaling using coarse predictors^8,15^. Oyama et al.^15^ used a super-resolution method based on generative adversarial networks to downscale precipitation with a scaling factor of 50. Nevertheless, the transferability of these methods to climate simulations has not been well explored^6,7,16^ and, among other issues, problems related to their extrapolation remain^17^.

Most previous studies have focused on surface temperature and precipitation^5,10,12,18^. Bano Medina et al.^12^ showed that CNNs offer a clear advantage for strongly nonlinear climate parameters such as precipitation, but only a minor benefit for temperature. They also suggested that CNNs could improve the reproducibility of extreme events. Although downscaling has been further applied to other climate variables such as wind speed^14^, the lack of reliable gridded observations over long periods often limits their broader application.

Downwelling shortwave radiation, usually called surface solar radiation, is a vital climate variable^19,20^. Due to its importance within agriculture and renewable energy sources, it is a crucial parameter for developing climate adaptation plans. To build long-term strategies for renewable energies such as photovoltaic (PV) systems, it is essential to evaluate how climate change impacts energy generation at a regional scale^21,22^. Renewable energy sources are expected to be central to transitioning to a low-carbon society^23^. However, meteorological conditions control their outputs. Solar radiation is modulated by cloud cover^24^ and aerosols^25^, and the efficiency of PV systems is affected by temperature and wind speed. Future climate change will modify these meteorological conditions, therefore affecting PV outputs^26^. With the rapid transition from fossil fuels to renewables such as solar PV, there is an increasing need to evaluate low solar output conditions that will significantly impact future PV infrastructure plans.

In this study, we applied a perfect prognosis approach to investigate several ML algorithms/approaches to downscale four meteorological variables (mean temperature, solar surface radiation, wind speed, and precipitation) in central Japan. In this way, we offered a rare opportunity to evaluate and compare the downscaling skills for these variables within the same context and approach. We focused on CNN, which has recently been applied to downscaling studies. Besides addressing the differences between CNN and linear models, we also faced the difference between CNN and classic nonlinear models. Overall, we found that CNN outperformed the other algorithms and allowed the generation of super-resolution climate scenarios.

On top of that, we demonstrated that the built CNN model, trained with observations, can be used to downscale CMIP6 simulations. We showed a reasonable agreement between downscaled CMIP6 variables and observations during the historical period and a consistent agreement between the future changes in the original low-resolution and high-resolution downscaled simulations. Recent attempts used CNN to downscale future CMIP6 simulations, but results were limited to temperature and precipitation^6^. We provided novel case studies for solar radiation and wind speed, which are rarely investigated primarily due to a lack of reliable long-term observations.

As an example of its potential application, we assessed future changes in PV power outputs. Showing the reliability of the downscaled PV potential, which arises from combining several variables, as compared to the PV potential of original low-resolution simulations, allowed us to contribute to exploring the issue related to the physical consistency of variables downscaled independently.

## Methods

**Figure 1:**
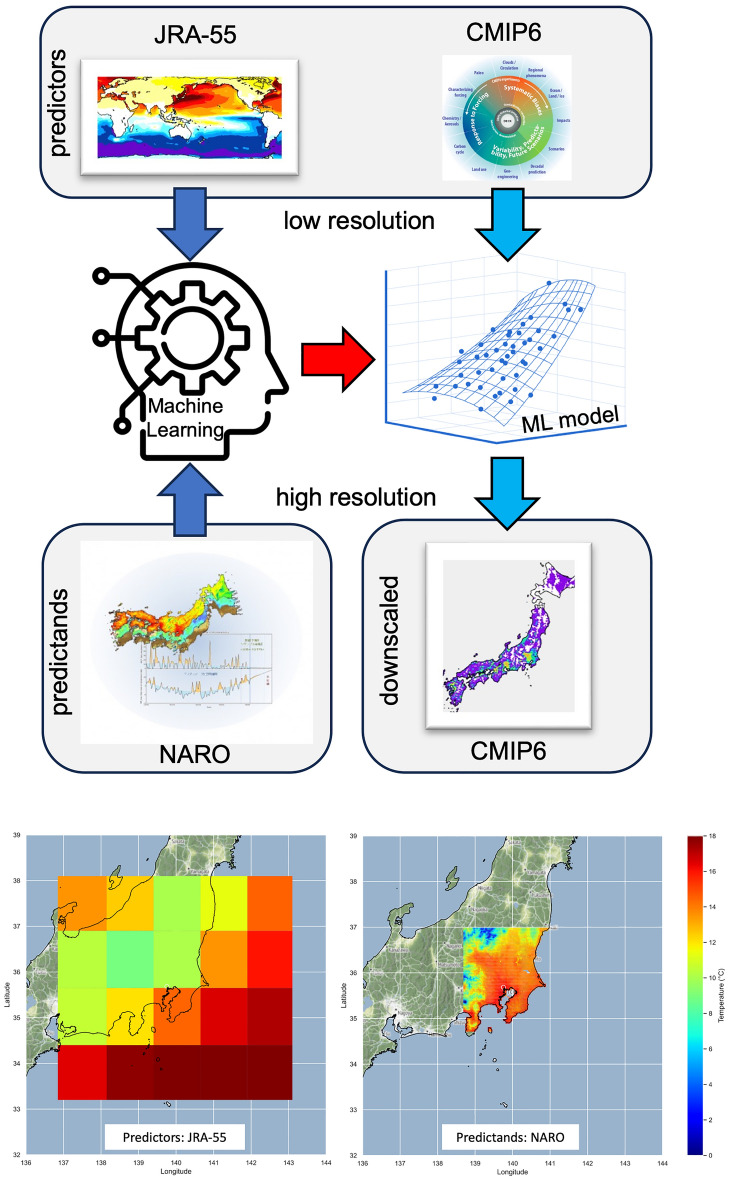
(Top) Summary of the approach adopted in this study. First, we used machine learning (ML) techniques and perfect predictors based on the Japan Meteorological Agency (JRA)-55 product to reproduce high-resolution observed predictands from the National Agriculture and Food Research Organization (NARO) dataset. Then, we used the ML model to downscale climate simulations. (bottom) Spatial domain of the low-resolution JRA-55 predictors and high-resolution NARO predictands. The predictor closest to each predictand grid cell was selected for artificial neural network (ANN), random forest (RF), and multiple linear regression (MLR) algorithms, and the entire predictor domain was used for a convolutional neural network (CNN) algorithm.

A summary of our approach is presented in Fig. 1. We first explored the accuracy, skills, and problems of four ML algorithms (e.g., CNN, ANN, RF, and MLR) during the calibration phase using perfect predictors and high-resolution observations of mean air temperature, surface solar radiation, precipitation, and wind speed as predictands. Following previous studies (e.g.,^5^), we adopted large-scale dynamical and thermodynamical variables at three different pressure levels as predictors. Three additional surface predictors were also included; we used the same set of predictors for all variables, except for one predictor (Table S1). All datasets were standardized to facilitate the training of the ML models.

We evaluated the contributions of nonlinear processes by comparing MLR with ANN and RF methods. Then, we determined the additional value provided by a CNN (which also included nonlinear activation functions) by examining the differences between the CNN and nonlinear algorithms. Finally, we focused on the transferability of the ML models to climate simulations, and we applied the best model (i.e., the CNN) to downscale climate simulations.

We used the Japan Meteorological Agency (JRA)-55 reanalysis^27^ product as a predictor because its spatial resolution (1.25° × 1.25°) is comparable to that of most climate simulations. As predictands, we used agro-meteorological grid square data from the National Agriculture and Food Research Organization (NARO), which has an original resolution of approximately 1 km with daily-scale data^28^. Then, we resampled the NARO datasets at various spatial resolutions ranging from 0.5° to 0.01°, which allowed downscaling results to be evaluated at various scaling factors. The bottom panels of Fig. 1 show the domain of the predictors and predictands used in this analysis. Most were available for the period 1980–2022 period, which was divided into training (1980–2004), test (2005–2014), and validation (2015–2022) periods. Due to the observational data availability (i.e., wind speed is available only from 2008) and other problems (i.e., an apparent discontinuity in surface solar radiation caused by interrupted observation in part of the study region), we were constrained to using 2015–2022 as the training period for wind speed and surface solar radiation. The test period was kept the same for all variables (note that we further assessed our setting using random sampled data recorded before 2005).

The ML analysis using an ANN (i.e., multi-layer perceptron regressor), RF, and MLR was based on the scikit-learn package, which is a Python module integrating a wide range of state-of-the-art ML algorithms (^29^; detailed information provided at: https://scikit-learn.org/stable/user_guide.html). We independently constructed the ML model for each grid cell, using the closest predictor cells. We used the TensorFlow package^30^ for the CNN.

Due to the time required to run some of the algorithms (e.g., ANN, see Fig. S1 and related discussion in section “Results”), we were unable to adopt a structured approach therefore, hyperparameter settings were based on a trial-and-error approach performed with the validation dataset and limited to the main hyperparameters. The ANN, RF, and CNN settings are provided in Table S2. For the CNN, we used a simple architecture^12^ with three layers (64, 32, and 16 features each), rectified linear unit (ReLu) activation functions in the hidden layers, a kernel size of 3 × 3, early stopping to prevent overfitting, and padding to maintain the original resolution of the predictors (Table S2). No evident improvements were achieved by using more sophisticated tuning (e.g., batch normalization and a dropout for the CNN). In this study, we report the mean ensemble of 10 members of the same ML model using different random initializations; this approach noticeably (slightly) improved the accuracy of the CNN (ANN and RF), but slightly degraded the reproduction of the extremes.

Following previous studies^26,31^, we focused our downscaling application on evaluating the solar energy potential under a future Shared Socioeconomic Pathway 585 (SSP585; i.e., a radiative forcing increase of 5–8.5 W/m^2^ by 2100) climate as the difference from the historical period. We defined the PV potential following Feron et al.^26^. Although PV energy yields depend on surface solar radiation, which are in turn driven by clouds and aerosols, PV outputs are also affected by cooling air temperatures and wind speeds, which generally improve PV cell performance. We used simulations from the Coupled Model Intercomparison Project sixth phase (CMIP6) by selecting the first member (r1i1p1f1) for both the historical and SSP585 scenarios of the Meteorological Research Institute Earth System Model Version 2.0 (MRI-ESM2-0,^32^) because it has proven to be reliable for Japan^33^ and has a resolution comparable with that of JRA-55. We present daily MRI data for 2005–2014 and 2055–2064 (further details of the method used can be found in the supplementary material).

## Results

CNNs work more efficiently than traditional ML algorithms, which are limited by having to train the ML model grid cell by grid cell. Figure S1 shows the time required to train each model over the investigated domain at a resolution of 0.025° × 0.025° (91 × 92 grid cells) for each algorithm using an NVIDIA A100 80 GB PCIe graphics processing unit (GPU). Although the analysis was affected by some limitations (e.g., ANN, RF, and MLR results are based on scikit-learn, whereas CNN results are based on TensorFlow), the CNN clearly performed much faster than the other models, by a factor of 2–3. The GPU memory was the bottleneck of the CNN, limiting the size of inputs and architecture that could be used^34^.

### Training and validation of the ML models with perfect predictors

**Figure 2:**
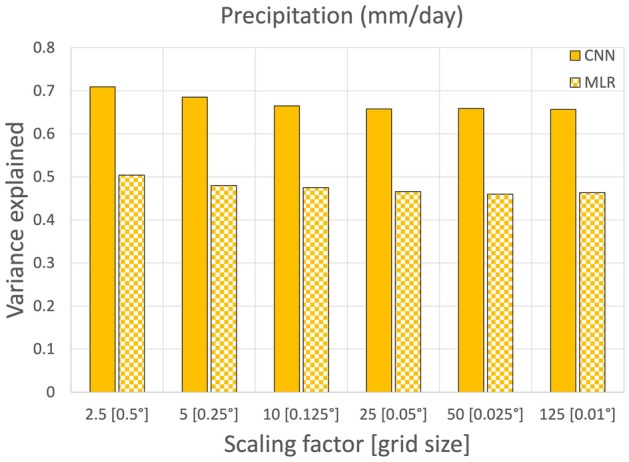
Explained variance of the downscaled precipitation versus observations averaged over the study domain during the test period for the MLR- and CNN-based models. We analyzed various scaling factors from 2.5 to 125, resulting in grid cells ranging from 0.5° to 0.01°.

The availability of gridded, very high-resolution observations in Japan at the country level, which are usually rare, motivated us to explore super-resolution downscaling^15^. The accuracy of the downscaling is expected to reduce from low to high resolution, although its extent is unknown. As precipitation is the most difficult predictand to reproduce, due to localized rainfall events resulting in a spatially clustered pattern, the changes with resolution can be better highlighted than other variables. Therefore, for the sake of simplicity, Fig. 2 shows the explained variance of the downscaled precipitation versus observations averaged over the investigated domain during the test period only for MLR and the CNN. By resampling the original dataset of the predictands, we performed downscaling at various scaling factors, resulting in a grid cell ranging from 0.5° to 0.01°. Overall, as expected, the explained variance slightly decreased with increasing grid resolution for scaling factors of 2.5–10. This behavior was similar for both MLR and the CNN. Surprisingly, downscaling with scaling factors from 10 to 125 resulted in a comparable explained variance, perhaps related to the original distances among the locations of the stations, usually ranging 10–20 km, which were conveyed to the gridded dataset. This result further encouraged us to explore super-resolution downscaling.

**Figure 3:**
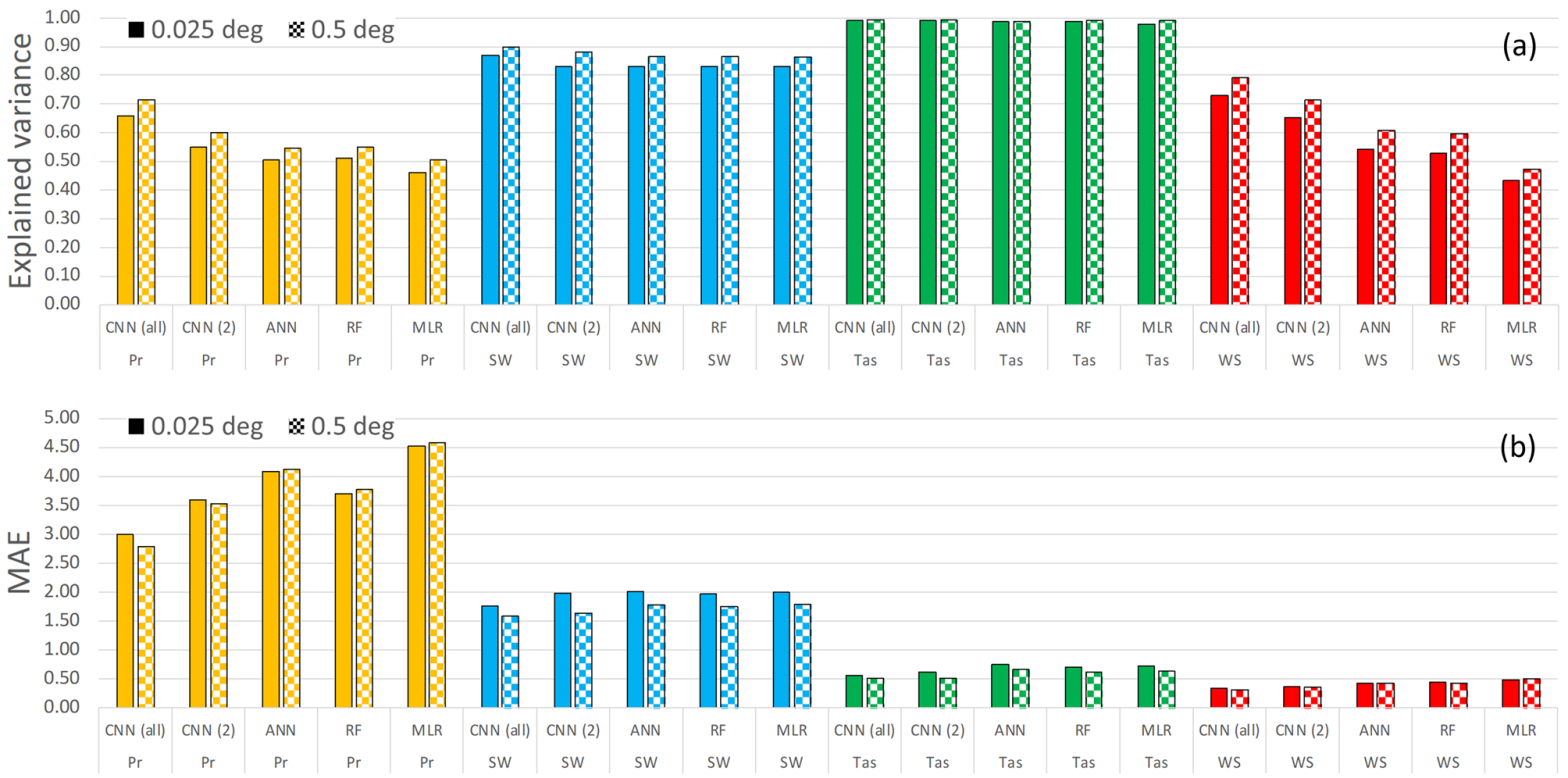
Comparison among ML algorithms (CNN, convolutional neural network; RF, random forest, ANN, artificial neural network and MLR, multiple linear regression) applied to the study domain for the test period 2005–2014: explained variance (**a**); mean absolute error (MAE) Pr: mm/day, Tas: °C, SW: MJ/m2/day, WS: m/s) (**b**). CNN(all) included all predictors, and CNN(2) included only two predictors (i.e., JRA55 SLP and the corresponding JRA55 variable to be downscaled). The downscaled outputs are presented at two resolutions (0.5° and 0.025°). Pr, precipitation; SSR, surface solar radiation; Tas, air temperature; WS, wind speed. See also Table S3.

Figure 3 summarizes comparisons among the ML algorithms, which were trained using 1980–2004 data and evaluated using 2005–2014 data, as described in section “Methods”. We introduced an additional CNN-based benchmark, called CNN(2). In this case, the CNN model included as variables only the regressor to be downscaled (i.e., temperature, solar radiation, wind speed, or precipitation) and sea level pressure (SLP) obtained from JRA-55. To compare results obtained at traditional and super-resolution, downscaled outputs were presented at two resolutions, 0.5° and 0.025° (Fig. 3 and Table S3). As shown by the largest explained variance and lowest mean absolute error (MAE) for all variables, the CNN was the algorithm with the best overall performance, and the accuracy was better for reduced than super-resolution downscaling. Nevertheless, the resolution only scaled the results, affecting all algorithms consistently.

Notably, the MLR, RF, and ANN provided comparable results for temperature and solar radiation, indicating the limited advantage of considering nonlinear relationships. In contrast, there was greater apparent advantage when downscaling precipitation and wind speed. Finally, because CNNs and ANNs (and the RF) can account for nonlinear relationships, their difference mainly resided in the convolutional approach of the CNN. Moreover, in most cases, CNN(2) (i.e., CNN based on only two regressors) already provided results comparable to or better than those of the other algorithms.

Although all algorithms produced similar biases for all variables, CNN was clearly superior for wind speed in terms of reproducing extreme events (Fig. S2, Table S3). More limited benefits were apparent for precipitation, solar radiation, and temperature. Because ML algorithms usually tend towards the mean and do not perform well for extremes (e.g.,^10^), the improved skill of the CNN was relevant.

As the CNN yielded the best scores, and the super-resolution method still provided a reliable downscaling, the analysis was limited to the CNN at a scaling factor of 50.

**Figure 4:**
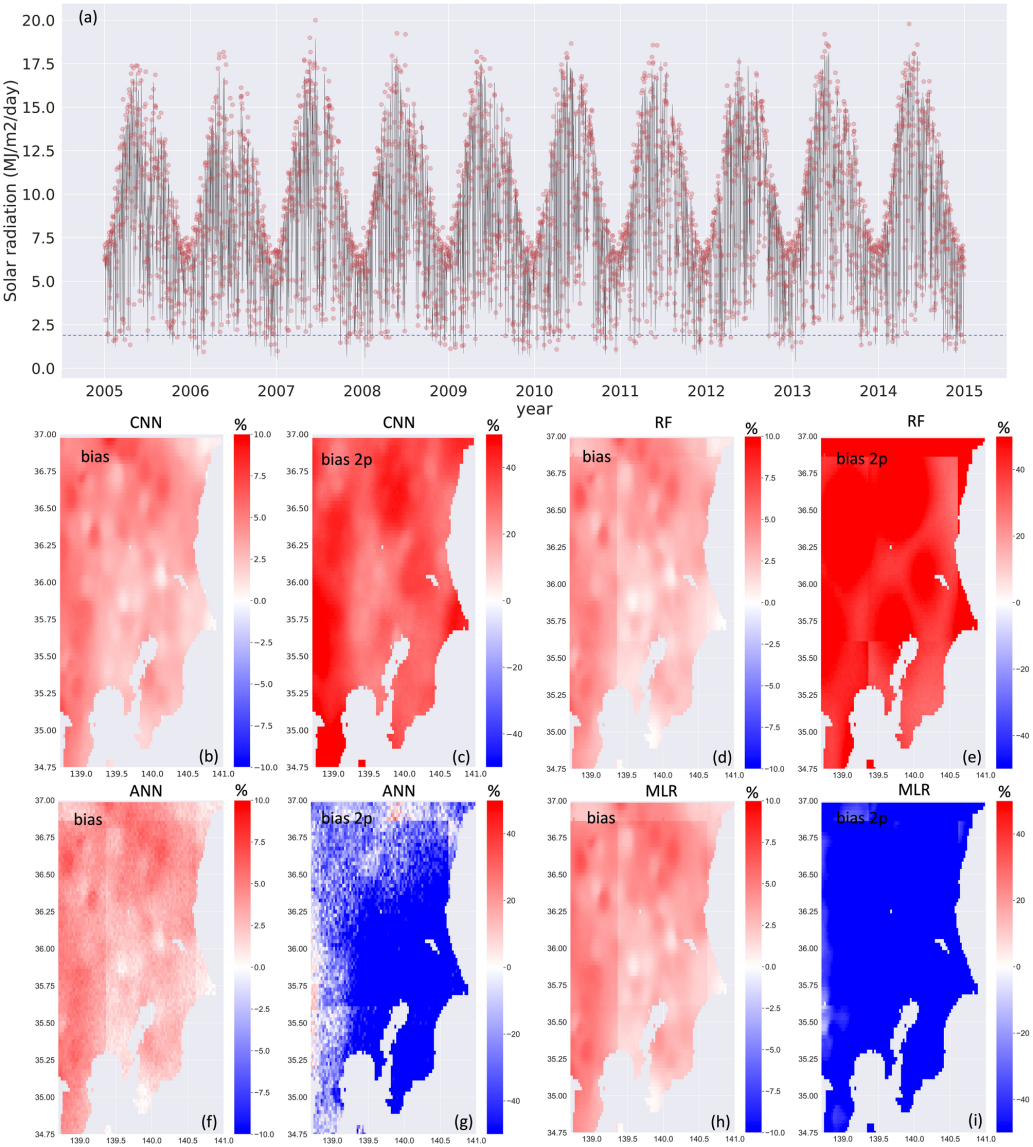
(Upper) Solar radiation averaged over the Kanto region during the test period for observations (black line) and CNN output (red circles). Horizontal dashed line in (**a**) indicates observed 2nd percentiles. (Lower) Spatial patterns of the (**a**, **d**, **f**, **h**) bias and (**c**, **e**, **g**, **i**) bias in 2nd percentiles produced by each algorithm. Despite similar bias produced by all algorithms (Table S3), the CNN better reproduced low solar output conditions, with domain-averaged values of 34.9%, − 47.3%, 48.5%, and − 104% for the CNN, ANN, RF, and MLR, respectively. Plots were generated using Python’s Matplotlib library, version 3.4.3, https://matplotlib.org/3.4.3/contents.html.

Figure 4a shows the downscaled and observed solar radiation averaged over the Kanto region during the test period. Although all algorithms rendered a similar bias (Fig. 4b, d, f, h), the signature of the regressor grid for the traditional algorithms (i.e., ANN, RF, and MLR) should be noted. As mentioned above, this spatial signature can be removed by taking the neighboring grid cells as additional predictors; however, this approach would result in a further computational burden that could be difficult to manage.

The CNN-based reconstructed solar radiation matched observations, although there was some overestimation under extreme high solar conditions (Fig. 4a and Table S3). Due to the importance of assessing low solar output conditions, which will significantly impact future PV infrastructure plans, we examined periods of low solar output by analyzing the bias in 2nd percentiles (Fig. 4c, e, g, i) produced by the different algorithms. The CNN better reproduced these low solar output conditions, with domain-averaged values of 34.9%, − 47.3%, 48.5%, and − 104% for the CNN, NN, RF, and MLR, respectively. This limited advantage could still be relevant for assessing regional-scale PV outputs.

To better evaluate the results, we examined the spatial patterns of metrics including the coefficient of determination, mean absolute error, bias, and bias of the 98th percentiles, standard deviation, and spatial autocorrelation (with respect to the central grid cell) for the downscaled variables over the test period (Figs. S3–S6) between CNN-based results and observations. The explained variance resulted in higher values for temperature (Fig. S3) and solar radiation (Fig. S4) and lower values for precipitation (Fig. S5) and wind speed (Fig. S6). As expected, the temperature and solar radiation (precipitation and wind speed) showed a limited (more significant) spatial heterogeneity. Overall, the general pattern of the multi-year mean and standard deviation was well reproduced in all datasets and the bias was usually small. Notably, it was difficult to reproduce the spatial autocorrelation and extreme conditions for precipitation.

The patches in the spatial pattern of some statistical parameters (e.g., for the MAE, panel b in Figs. S3–S6) were due to limitations of the original high-resolution dataset of predictands. Although this is the best available dataset to date in terms of temporal and spatial resolution, as an observational dataset derived from a dense network of surface stations, it nonetheless required the interpolation of observations over a regular grid (see also Fig. 2). This is reflected in the spatial pattern.

### Assessment of future changes in PV power outputs

**Figure 5:**
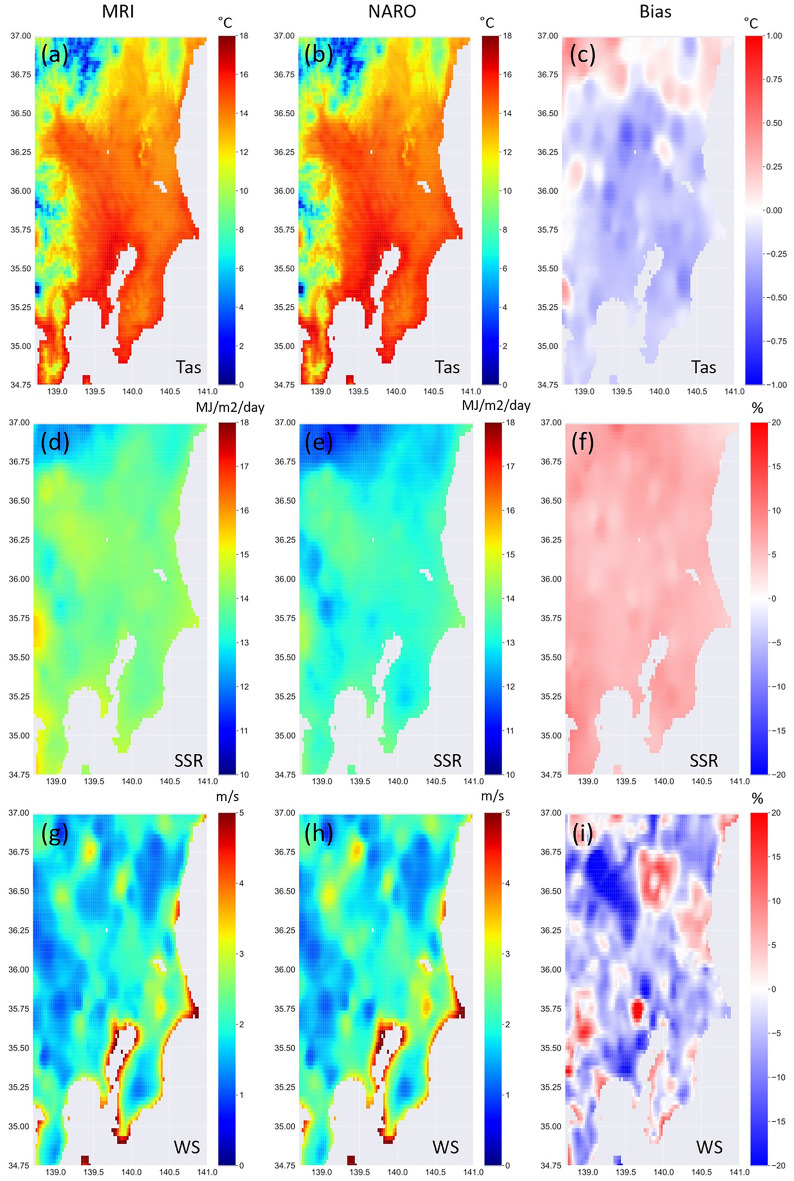
CNN-based downscaled MRI simulations (left column), observations (central column), and resultant bias (right column) for air temperature (top), surface solar radiation (middle), and wind speed (bottom) during the historical period (2005–2014). Tas, air temperature; SSR, surface solar radiation; WS, wind speed. Plots were generated using Python’s Matplotlib library, version 3.4.3, https://matplotlib.org/3.4.3/contents.html.

Figures S3–S6 show that the main issues of this downscaling exercise were related to precipitation (see also results for wet days in Fig. S7). Although the average rainfall was well reproduced (Fig. S5), statistics showed worse results than the other parameters. Indeed, additional analysis (Fig. S7) showed that while the fraction of wet days was overestimated, the precipitation amounts were underestimated. This suggests the necessity of developing a specific approach to precipitation. Therefore, we restricted the application of the CNN to climate simulations of temperature, surface radiation, and wind speed. We adopted the MRI climate model, which has been demonstrated to be reliable for Japan^33^ and has a similar resolution to JRA-55. Figure 5 shows the patterns of mean observed and simulated variables and their respective biases averaged over the historical period 2005–2014. It should be noted that as the CMIP6 simulations are not synchronized with observations, we did not show other statistics such as correlation and mean absolute error, which could be misleading and worse than the corresponding values in Figs. S3–S6. Overall, the observed variables were reproduced satisfactorily, with the bias of air temperature only marginally negative and that of surface solar radiation slightly positive (i.e., by a few percent). The wind speed results were also reasonable, showing overall slightly negative bias with some positive patches.

**Figure 6:**
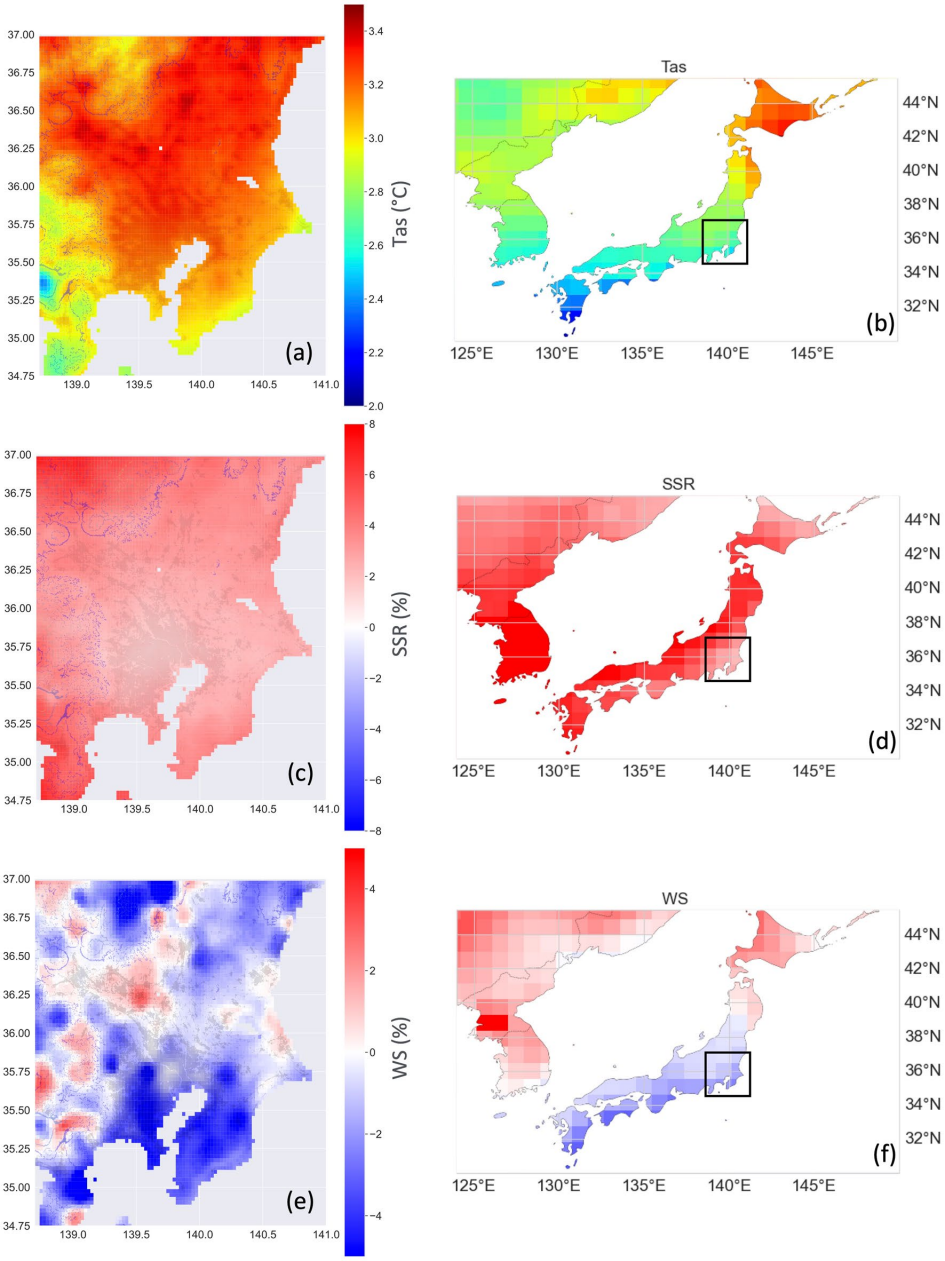
Time series differences for Shared Socioeconomic Pathway 585 (SSP585; i.e., radiative forcing increase of 5–8.5 W/m^2^ by 2100) (2055–2064) minus historical (2005–2014) based on original Meteorological Research Institute (MRI) data (right column) and corresponding downscaled variables at 0.025° (left column) as follows: (**a**, **b**) air temperature, (**c**, **d**) surface solar radiation, and (**e**, **f**) wind speed. In (**a**, **c**, **e**), 500, 1000, 1500, and 2000 m a.s.l. contours indicate topography. Black dots indicate built-up urban areas retrieved from a Japan Aerospace Exploration Agency (JAXA) high-resolution land-cover map. Plots were generated using Python’s Matplotlib library, version 3.4.3, https://matplotlib.org/3.4.3/contents.html.

The differences between future and historical downscaled and original time slice MRI simulations are shown in Fig. 6. The downscaled MRI output (left column) indicates that temperature would increase by 2–3 °C in the study region, with a southerly gradient running from the most significant increase in the northeast to the lowest in the southwest. This result agrees with the corresponding changes in the original MRI simulations (right column), although temperature resulted in slightly higher values. The built-up urban area is also shown as a reference. These downscaled temperature changes do not account for the contributions of future variation due to urbanization. The inclusion of observations influenced by Tokyo’s heat island in the training data resulted in patterns of future high-resolution temperature changes that were driven by present urbanization.

Downscaled surface solar radiation is also predicted to increase around Japan under a future climate, and both its magnitude and pattern appear to match the original MRI simulations. Finally, low-resolution MRI wind speed simulations are expected to decrease in the study region, whereas high-resolution data, although generally decreasing, was also enhanced in some limited areas. In general, the high-resolution MRI simulations indicated that the topographic pattern of the region would determine the extent of future changes and that, among the investigated variables, these variations remained coherent. Overall, the general patterns agreed with those of previous studies based on ensemble CMIP5 models showing future increases in solar radiation due to circulation trends causing cloud cover decreases and reduced wind speeds around Japan^35^.

The Kanto region is dominated by ocean coastlines in the south and east, and by mountains in the north and west. Some original low-resolution grid cells contained both land and ocean areas, representing the average of both environments. In contrast, the CNN model was trained only with observations located on land. Similarly, there were minor discrepancies in wind speed between high- and low-resolution datasets in mountain areas where GCMs cannot reproduce the complex topography.

**Figure 7:**
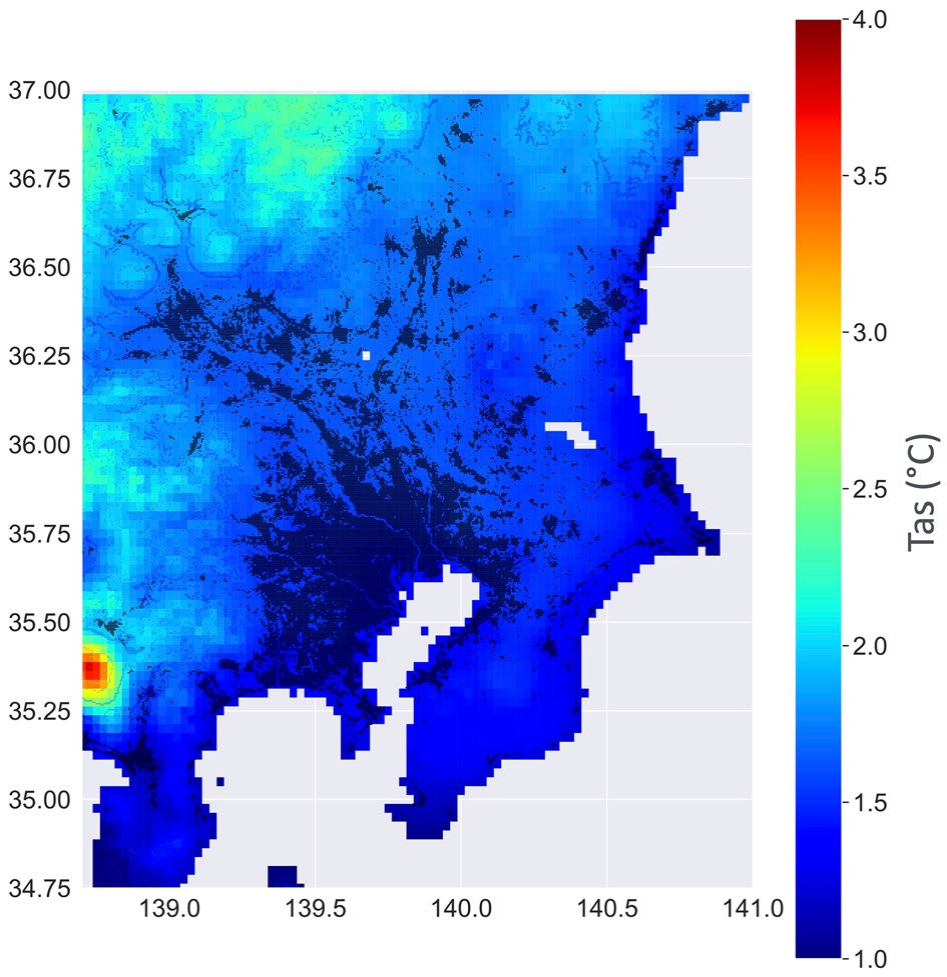
MRI time series differences for temperature (°C) as shown in Fig. 6a, except for winter (DJF) months. Mount Fuji’s location corresponds to the high patch in the southwest. Plots were generated using Python’s Matplotlib library, version 3.4.3, https://matplotlib.org/3.4.3/contents.html.

A closer examination of the seasonal temperature variation further supported the reliability of our downscaling. Previous studies based on dynamic downscaling approaches showed a more significant warming rate in Japan’s mountainous regions than in plains in winter under extreme climate scenarios (e.g.,^4,36^). This result was expected, mainly due to the increased surface albedo caused by reduced snow cover. Our ML-based downscaling model cannot reproduce the details of the future pattern of temperature changes over complex terrains. Nevertheless, it is remarkable that in agreement with Sasaki et al.^36^, future temperature variations resulted more significant in mountainous areas in winter (Fig. 7).

**Figure 8:**
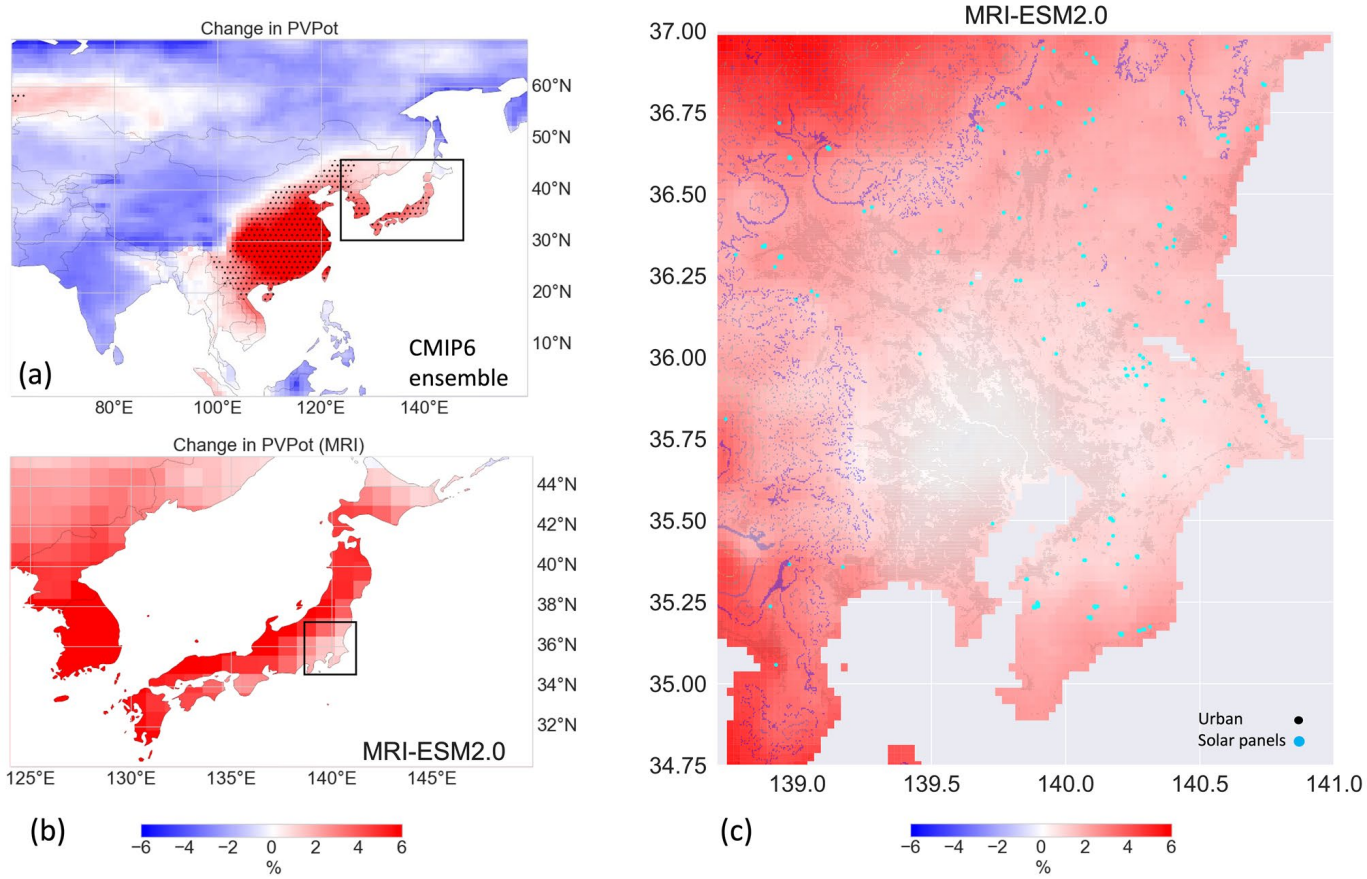
(**a**) Photovoltaic (PV) potential in 2055–2064 (SSP585) minus PV potential in 2005–2014 (historical) as rendered by the CMIP6 ensemble in East Asia, (**b**) MRI simulation at the original resolution over Japan, and (**c**) downscaled MRI simulation (0.025°) over the Kanto region. The stippled area in (**a**) indicates significant changes. In (**c**), 500, 1000, 1500, and 2000 m a.s.l. contours indicate topography. Blue dots indicate large PV deployments and black dots indicate urban built-up areas retrieved from a JAXA high-resolution land-cover map. Plots were generated using Python’s Matplotlib library, version 3.4.3, https://matplotlib.org/3.4.3/contents.html.

Figure 8 shows the differences between future and historical time slice simulations of PV potential^26^. To qualitatively assess the downscaling results and place the results of a single model into the context of a CMIP6 ensemble, Fig. 8a shows the PV potential change arising from a five-model ensemble that covers the wide uncertainty of the CMIP6 ensemble^33^ and Fig. 8b shows the changes in low-resolution MRI PV potential over Asia and Japan. Recent studies based on a different CMIP6 ensemble (e.g.,^31^) showed that global PV potential is expected to decrease under a future climate, except in East Asia and part of South America. Figure 8a confirms these previous findings and depicts a clear pattern, with a significant increase in PV potential in eastern China, Korea, and Japan and a general decrease in the rest of Asia. The MRI model followed this general behavior, showing a 6–7% increase in PV potential, particularly in Korea and western Japan (Fig. 8b). Conversely, in Kanto, there were limited enhancements, with the largest increases mainly in the north and west, followed by the south, with minimal changes in the central area. This overall trend found in the raw MRI data was consistent with the downscaled PV potential presented in Fig. 8c. Overall, we noted small changes in urbanized areas, with more significant enhancements in regions dominated by mountains (north) and around Mount Fuji (southwest). Moreover, PV potential was generally higher in the Izu and Boso Peninsulas.

To contextualize these, Fig. 8c shows the main regions with PV deployments, as determined by the Japan Aerospace Exploration Agency (JAXA) high-resolution land-cover map (where PVs are generically indicated as solar panels). Most PV systems were installed along the Pacific side of the Kanto region, in Chiba and Ibaraki Prefectures, where the topography is homogeneous and flat. This area will be marginally affected by future climate change. Although the areas in the North and South-West of Kanto will show a somewhat higher PV potential in the future, they are primarily coincident with mountain regions with complex topography. Currently, there is an increasing number of solar plants being built in mountain areas in Japan, essentially due to the lack of available flat land^37^ and increasing measures to limit the deployment of solar parks in agricultural areas. Nevertheless, mountain regions are not optimal for installing solar plants due to nature conservation issues and land use impact. Moreover, solar PVs are installed in mountainous areas after deforestation and land reclamation^38^. Therefore, the associated risk (e.g., landslides) caused by frequent extreme events must be considered.

On the other hand, the current legislation of the Japanese capital's is moving in the opposite direction, i.e., all new houses in Tokyo built by large-scale homebuilders after April 2025 must install PV roof systems to cut household carbon emissions. Potentially, by shading the roofs, more extensive use of PV roof systems could contribute to easing the severe Tokyo's urban heat island effects^39^. Nevertheless, recent findings questioned the cooling effect of urban PV systems, pointing out that several complex mechanisms are at play^40^.

## Discussions

In this study, taking advantage of the availability of gridded high-resolution observations, we downscaled four meteorological variables over central Japan. In addition to commonly investigated variables such as mean air temperature and precipitation, we downscaled wind speed and solar surface radiation, which have rarely been investigated due to the scarcity of long-term, reliable high-resolution observational datasets.

We trained and evaluated four ML models, based on CNN, ANN, RF, and MLR algorithms, with observations. Overall, the downscaling performance of the four variables followed the order of: temperature > solar surface radiation > wind speed > precipitation. This ranking emerged despite having trained solar radiation and wind speed with shorter datasets than that of rainfall.

We found that the CNN outperformed the other algorithms for all variables. The CNN improved downscaling performance mainly for wind speed and precipitation, followed by solar radiation and temperature. Overall, we detected significant CNN-related improvement over traditional nonlinear (ANN and RF) and linear (MLR) algorithms. Because the CNN included non-linear activation functions, we quantified the advantage of the CNN’s convolutional approach over traditional algorithms applied to each grid cell independently.

After assessing the dependence of the accuracy of the downscaled results on the scaling factor, we focused on a super-resolution CNN method with a scaling factor of 50, which resulted in grid cells of 0.025°. We confirmed the difficulty of downscaling precipitation, but obtained acceptable accuracy for wind speed, solar radiation, and temperature, with explained variances of 0.66, 0.73, 0.87, and 0.99, and mean absolute errors of 3.00 mm/day, 0.333 m/s, 1.76 MJ/day, and 0.56 °C, respectively, with negligible bias (except for precipitation). These results were marginally weaker than those obtained at smaller scaling factors (Table S3 and Figs. 2, 3).

An additional advantage of CNN was its better representation of extreme events. Japan's electricity system is transitioning rapidly to renewable energy. According to the strategic energy plan, renewable energy will cover 36–38% of the power supply (14–16% of solar PV) by 2030^41^. With the anticipated increase of PV penetration levels into the electricity grid, weather-induced variability in PV power output is expected to negatively impact grid stability due to electricity generation fluctuations^21^. This scenario increases the urgency of determining the probability and duration of periods of meager solar output. Within this context, CNN should be considered when assessing renewables at a regional scale.

As an example of its potential application, we assessed future changes in PV power outputs, which are central to transitioning to a decarbonized society but are also modulated by future climate change and weather variability. Although PV energy yields depend on solar radiation, driven by clouds and aerosols, PV outputs are also affected by air temperature and wind speed, with cooler conditions generally improving the PV cell performance. Therefore, we used the CNN model to downscale MRI historical and future climate simulations, resulting in an appropriate reproduction of temperature, surface solar radiation, and wind speed observations during the historical period. After building super-spatial resolution downscaling scenarios, MRI simulations revealed that regional topographic patterns determined the extent of future changes in these variables and that these changes were coherent with each other. Notably, high-resolution MRI temperature data revealed more significant warming rates in mountainous regions than in plains during winter. This finding is consistent with previous studies that adopted a dynamical downscaling approach^36^. Nevertheless, the stationarity assumption that drives ML-based methods will probably fail in other mountainous areas where warming could modify snow regimes and land–atmosphere coupling mechanisms differently. Overall, ML-based downscaling is not expected to compete with regional models but can improve results based on traditional downscaling methods by adding further spatial and temporal information.

Although solar irradiance, wind speed, and temperature change during the day, as in previous studies^26,42,43^, we used daily estimates rendered by a climate model simulation for computing daily estimates of the PV potential. High-temporal-resolution solar downscaling would allow more insight into PV-related applications. However, many climate models do not offer sub-daily resolution; if so, some variables are sometimes lacking. Moreover, accurate surface observations of solar radiation are usually sparse spatially and temporally. As a result, the high-resolution observational dataset we used to train the ML model is at a daily level.

The actual values of PV potential could differ somewhat from those we computed by a given amount. Still, changes in PV potential are expected to be coherent as this amount will affect the results similarly in both the historical and the future periods. On the other hand, analyzing the PV potential during a given period and its eventual comparison with real-world observations could be more challenging to carry out this way.

Overall, our analysis of PV power outputs confirmed the results of previous studies that suggested generally increased solar power output in Japan^22,35^. Therefore, our results confirm the reliability of the CNN method for producing super-resolution climate scenarios and will enable energy planners to anticipate the effects of future weather variability.

### Supplementary Information

Supplementary Information.

## Data Availability

The datasets generated and/or analyzed during the current study are available from the corresponding author on reasonable request.
