# Author Correction: Exploring super-resolution spatial downscaling of several meteorological variables and potential applications for photovoltaic power

**DOI:** 10.1038/s41598-024-64055-y

**Published:** 2024-06-07

**Authors:** Alessandro Damiani, Noriko N. Ishizaki, Hidetaka Sasaki, Sarah Feron, Raul R. Cordero

**Affiliations:** 1 NIES, Tsukuba, Japan; 2 University of Groningen, Leeuwarden, The Netherlands; 3 Universidad de Santiago de Chile, Santiago, Chile

Correction to: *Scientific Reports* 10.1038/s41598-024-57759-8, published online 27 March 2024

The original version of this Article contained an error in the legend of Figure 7.

“MRI time series differences for temperature (°C) as shown in Fig. 6a, except for winter (DJF) and summer (JJA) months, which are shown in (a) and (b), respectively. Mount Fuji’s location corresponds to the high (low) patch in the southwest in panel a (b). Plots were generated using Python’s Matplotlib library, version 3.4.3, https://matplotlib.org/3.4.3/contents.html.”

now reads:

“MRI time series differences for temperature (°C) as shown in Fig. 6a, except for winter (DJF) months. Mount Fuji’s location corresponds to the high patch in the southwest. Plots were generated using Python’s Matplotlib library, version 3.4.3, https://matplotlib.org/3.4.3/contents.html.”

The original Article has been corrected.

