# Supplementary material for: Exploring super-resolution spatial downscaling of several meteorological variables and potential applications for photovoltaic power
Source: Sci Rep. 2024 Mar 27;14:7254. doi: 10.1038/s41598-024-57759-8 (PMC10973502; doi:10.1038/s41598-024-57759-8)
Supplement: Supplementary file 1 — Supplementary Information. [file 41598_2024_57759_MOESM1_ESM.pdf]

SUPPLEMENTARY MATERIAL

Exploring super-resolution spatial downscaling of several meteorological variables and potential applications for photovoltaic power

Alessandro Damiani[1,*], Noriko N. Ishizaki[1], Hidetaka Sasaki[1], Sarah Feron[2,3], Raul R. Cordero[3]

[1]NIES, Tsukuba, Japan

[2]University of Groningen, Leeuwarden, The Netherlands

[3]Universidad de Santiago de Chile, Santiago, Chile

*damiani.alessandro@nies.go.jp

Figures

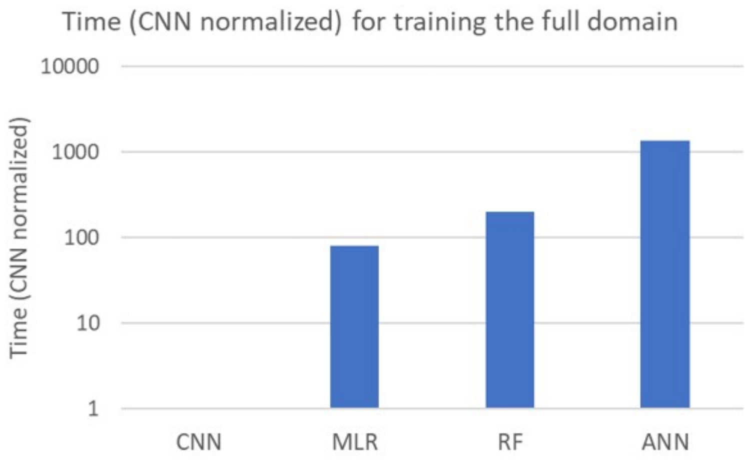

Fig. S1. Time required to train each of four ML-based models over the investigated domain, with a resolution of 0.025° (91 × 92 grid cells).

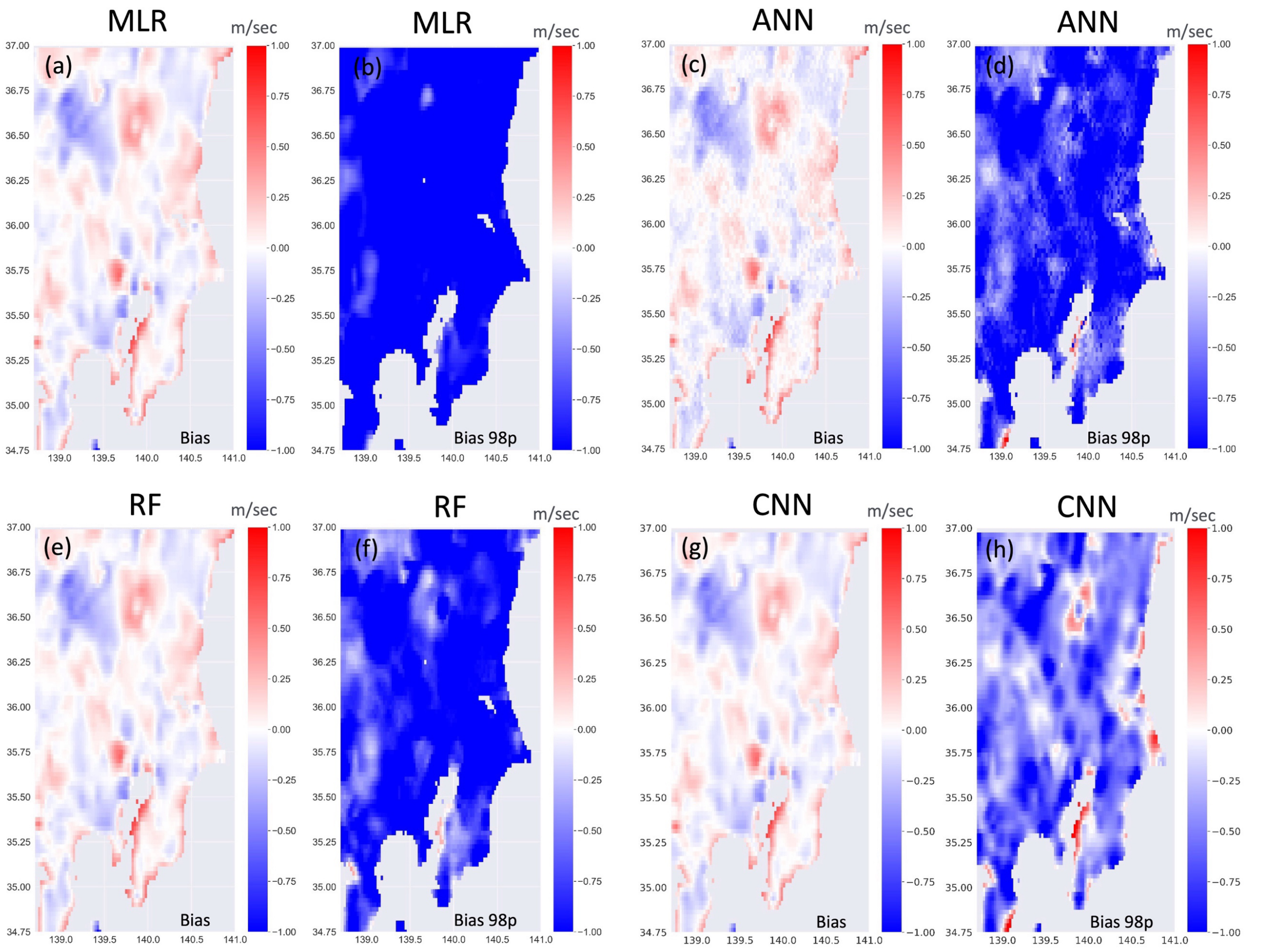

Fig. S2 Bias and bias of the 98th percentile for wind speeds during the test period 2005–2014, for models based on the MLR, RF, ANN, and CNN algorithms (spatial domain-averaged values are provided in Table 3).

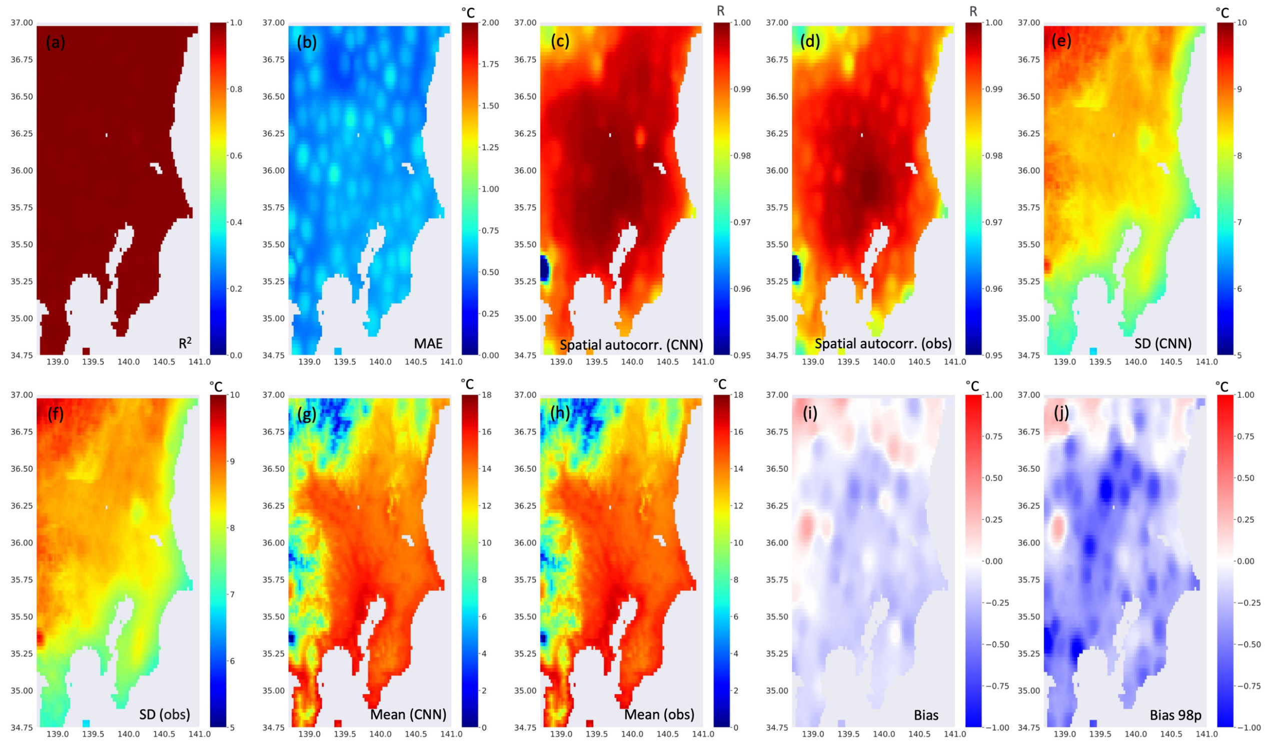

Fig. S3. CNN-based downscaling results (and observations) averaged over the test period (2005–2014) for temperature downscaled to 0.025° × 0.025° grid cell resolution using a scaling factor of 50 (cf. Figs. 2 and 3). The training period was 1980–2004. R^2, explained variance (a); MAE, mean absolute error (b); CNN spatial autocorrelation (c); observed spatial autocorrelation (d); CNN standard deviation (e); observed standard deviation (f); observed mean (g); CNN mean (h); bias (i); bias (98th percentile) (j).

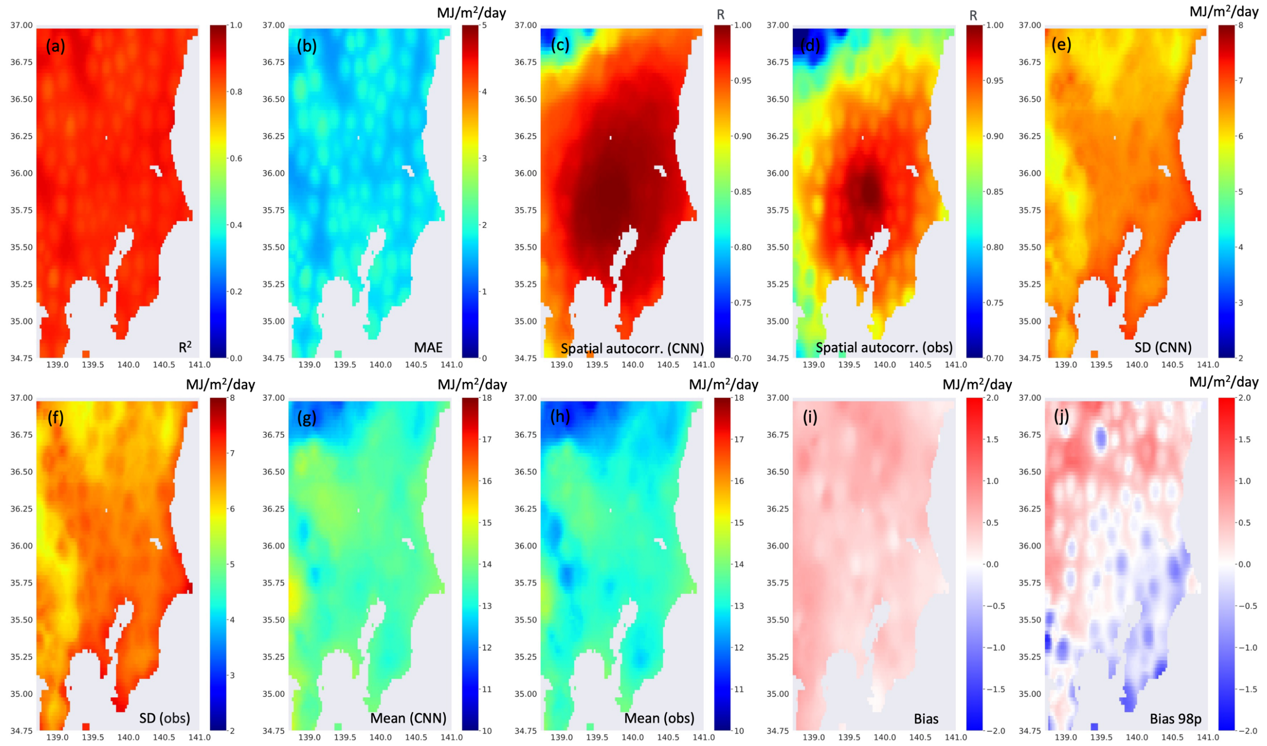

Fig. S4. CNN-based downscaling results (and observations) averaged over the test period (2005–2014) for solar radiation downscaled to 0.025° × 0.025° grid cell resolution using a scaling factor of 50 (cf. Figs. 2 and 3). The training period was 2015–2022. R^2, explained variance (a); MAE, mean absolute error (b); CNN spatial autocorrelation (c); observed spatial autocorrelation (d); CNN standard deviation (e); observed standard deviation (f); observed mean (g); CNN mean (h); bias (i); bias (98th percentile) (j).

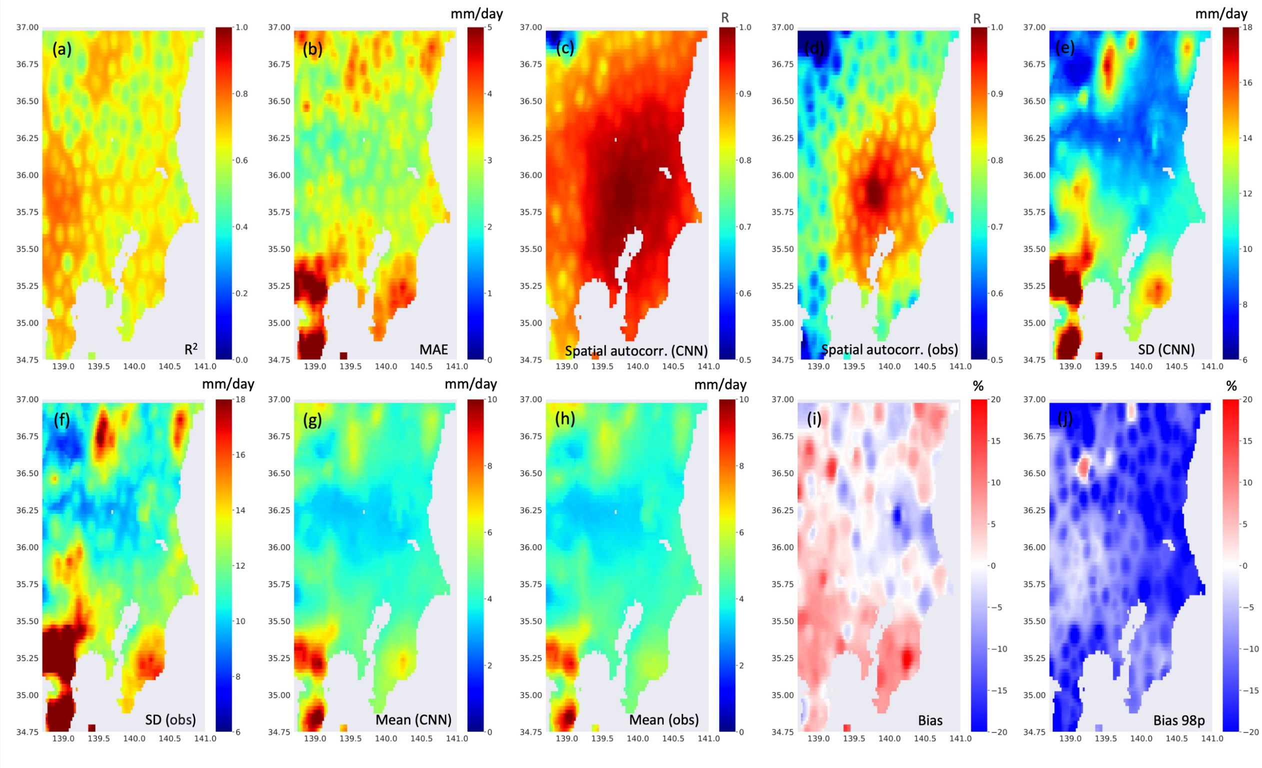

Fig. S5. CNN-based downscaling results (and observations) averaged over the test period (2005–2014) for precipitation downscaled to 0.025° × 0.025° grid cell resolution using a scaling factor of 50 (cf. Figs. 2 and 3). The training period was 1980–2004. R^2, explained variance (a); MAE, mean absolute error (b); CNN spatial autocorrelation (c); observed spatial autocorrelation (d); CNN standard deviation (e); observed standard deviation (f); observed mean (g); CNN mean (h); bias (i); bias (98th percentile) (j).

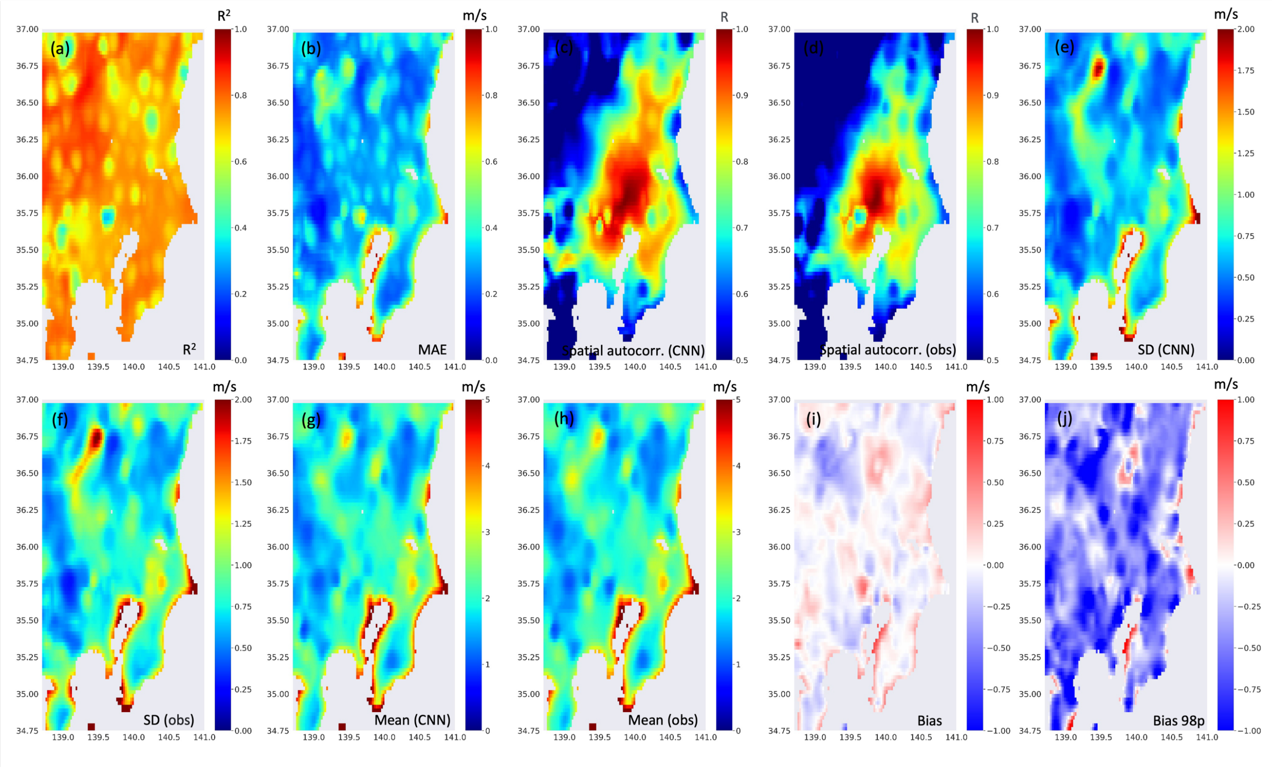

Fig. S6. CNN-based downscaling results (and observations) averaged over the test period (2005–2014) for wind speed downscaled to 0.025° × 0.025° grid cell resolution using a scaling factor of 50 (cf. Figs. 2 and 3). The training period was 2015–2022. R^2, explained variance (a); MAE, mean absolute error (b); CNN spatial autocorrelation (c); observed spatial autocorrelation (d); CNN standard deviation (e); observed standard deviation (f); observed mean (g); CNN mean (h); bias (i); bias (98th percentile) (j).

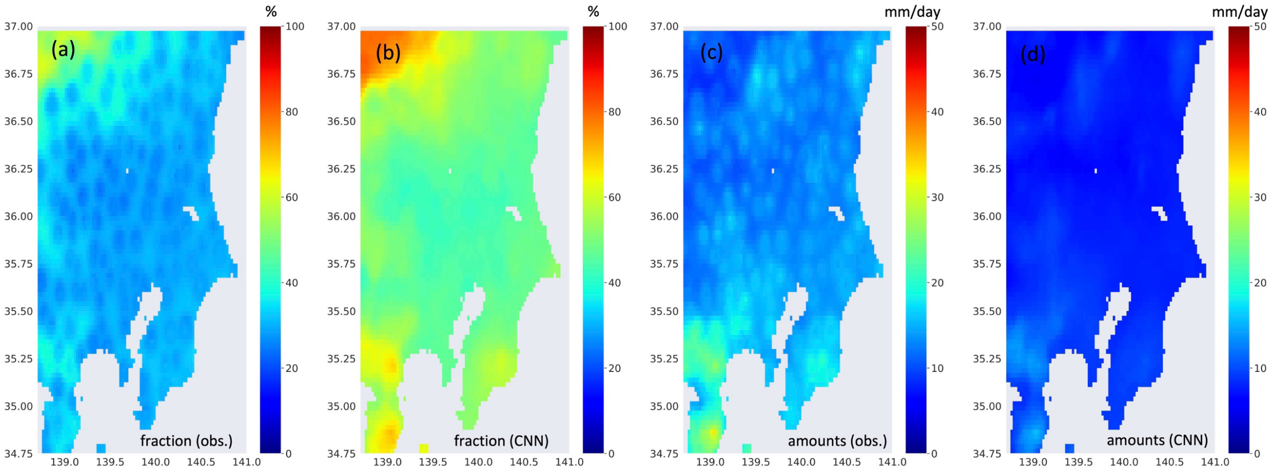

Fig. S7. Comparison of observed and downscaled (CNN-based) precipitation for wet days (precipitation > 1 mm/day) averaged over the test period (2005–2014). Fraction of wet days observed (a) and downscaled (b); precipitation amounts in wet days observed (c) and downscaled (d).

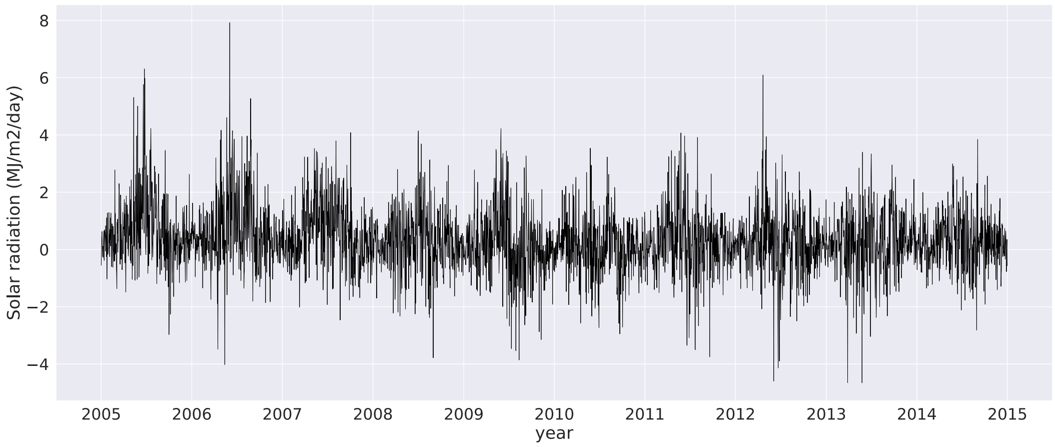

Fig. S8. Solar radiation averaged over the Kanto region during the test period (2005–2014), as in Fig. 4a, except for the difference (CNN output minus observations).

Tables

High-resolution NARO Predictand	Tas	SSR	Pr	WS
Low-resolution JRA-55 Predictors	U at 850, 700, 500 hPa	U at 850, 700, 500 hPa	U at 850, 700, 500 hPa	U at 850, 700, 500 hPa
	V at 850, 700, 500 hPa	V at 850, 700, 500 hPa	V at 850, 700, 500 hPa	V at 850, 700, 500 hPa
	RH at 850, 700, 500 hPa	RH at 850, 700, 500 hPa	RH at 850, 700, 500 hPa	RH at 850, 700, 500 hPa
	ZG at 850, 700, 500 hPa	ZG at 850, 700, 500 hPa	ZG at 850, 700, 500 hPa	ZG at 850, 700, 500 hPa
	T at 850, 700, 500 hPa	T at 850, 700, 500 hPa	T at 850, 700, 500 hPa	T at 850, 700, 500 hPa
	Tas	Tas	Tas	Tas
	SLP	SLP	SLP	SLP
	SSR	SSR	Pr	WS

Table S1. List of Japan Meteorological Agency (JRA)-55 low-resolution predictors and high-resolution National Agriculture and Food Research Organization (NARO) predictands. Pr, precipitation; RH, relative humidity; SLP, sea-level pressure; SSR, surface solar radiation; T, temperature; Tas, air temperature; U, eastward wind component; V, northward wind component; WS, wind speed; ZG, geopotential height.

Algorithm name	MLR	ANN	RF	CNN
Main settings	default	epochs = 500	n_estimators=100	epochs = 500
		optimizer = adam	loss=mean squared error	optimizer = adam
		activation = relu	max_depth=None	activation = relu
		early stopping	min_samples_leaf=1	early stopping
		shuffle=True		shuffle=True
				three layers
				kernel size 3×3
				padding
				loss=mean squared error

Tab. S2. Main hyperparameter settings applied to the four machine learning (ML) algorithms.

Variable<br>Size	Type	R2<br>0.025	R2<br>0.50	MAE<br>0.025	MAE<br>0.50	Bias<br>0.025	Bias<br>0.50	Bias_p98<br>0.025	Bias_p98<br>0.50
Pr	CNN(all)	0.660	0.715	3.000	2.792	-2.600	-2.480	-16.900	-13.480
	CNN(2)	0.550	0.601	3.600	3.530	5.200	3.044	-17.700	-14.770
	ANN	0.505	0.545	4.080	4.130	-1.910	0.034	-31.190	-18.690
	RF	0.510	0.550	3.710	3.774	8.200	8.2	-21.070	-21.070
	MLR	0.460	0.504	4.530	4.589	-0.080	-1.27	-24.470	-30.900
SW	CNN(all)	0.870	0.898	1.760	1.580	3.600	0.2	1.200	-1.010
	CNN(2)	0.830	0.880	1.990	1.634	4.000	0.347	-1.200	-0.263
	ANN	0.830	0.866	2.009	1.776	3.400	0.46	-4.300	-0.865
	RF	0.831	0.865	1.970	1.752	3.170	0.411	-1.897	-0.222
	MLR	0.830	0.864	2.000	1.799	3.600	0.472	-5.440	-1.114
Tas	CNN(all)	0.992	0.994	0.560	0.512	-0.015	-0.241	-0.190	-0.499
	CNN(2)	0.990	0.993	0.610	0.513	-0.130	-0.15	-0.480	-0.279
	ANN	0.987	0.989	0.744	0.661	-0.150	-0.17	-0.490	-0.454
	RF	0.988	0.990	0.710	0.622	-0.14	-0.14	-0.44	-0.409
	MLR	0.980	0.990	0.720	0.632	0.070	-0.139	0.060	-0.229
WS	CNN(all)	0.731	0.792	0.331	0.305	0.002	-0.019	-0.429	-0.444
	CNN (2)	0.652	0.714	0.372	0.349	-0.037	0.004	-0.492	-0.349
	ANN	0.542	0.609	0.431	0.422	-0.009	-0.007	-0.879	-0.768
	RF	0.528	0.596	0.433	0.420	-0.005	-0.006	-0.966	-0.880
	MLR	0.433	0.473	0.481	0.491	-0.003	-0.006	-1.447	-1.380

Table S3. Statistics of the employed ML algorithms, which were trained using 1980–2004 data (except wind speed and surface solar radiation data, see Section 2) and evaluated using 2005–2014 data. The results show the average over the whole study domain during the test period of 2005–2014. Results are expressed as absolute values (i.e., Pr: mm/day, Tas: °C, SW: MJ/m^2/day, WS: m/s), except for the bias (%) and 98th percentile bias (%) of Pr.

Computation of photovoltaic (PV) potential

The selected method to compute the changes between historical and future PV potential is based on our previous work (Feron et al., 2021).

The PV potential can be defined as the fraction of the power output under standard conditions that a PV module may display in the field

PV pot = Pr I/I(stc)

I(stc) = solar irradiance applied to the module under standard test conditions (1,000 W/m2);

I = solar irradiance imposing on the PV modules in the field;

Pr = performance ratio accounting for the effect of the cell temperature, T(cell), on its efficiency.

Then, Pr can be calculated as follows

Pr = 1 – γ (T(cell) – T(stc))

T(stc) = cell temperature under standard test conditions and a higher cell temperature leads to poorer performance. Finally, T(cell) would depend on both temperature and wind speed (Feron et al., 2021; Jerez et al., 2015; Chenni et al., 2007), with wind favoring cooling of the PV module, in turn leading to a lower T(cell). Further details and references can be found in Feron et al. (2021).
